# Varied treatment pathways with no defined treatment sequencing in patients with generalized pustular psoriasis: A claims-based study

**DOI:** 10.1016/j.jdin.2023.11.007

**Published:** 2023-12-16

**Authors:** Steven R. Feldman, Ran Gao, Rhonda L. Bohn, Stephani Gray, Sabrina E. Walton, Anouk Déruaz-Luyet, Jashin J. Wu

**Affiliations:** a Department of Dermatology, Wake Forest School of Medicine, Winston-Salem, North Carolina; b Boehringer Ingelheim Pharmaceuticals Inc., Ridgefield, Connecticut; c Bohn Epidemiology, Boston, Massachusetts; d Boehringer Ingelheim International GmbH, Ingelheim, Germany; e Department of Dermatology, University of Miami, Miller School of Medicine, Miami, Florida

**Keywords:** autoinflammatory disease, claims data, cohort study, generalized pustular psoriasis, observational study, treatment patterns

*To the Editor:* Generalized pustular psoriasis (GPP) is a chronic, rare, life-threatening skin disease, characterized by recurrent flares of widespread sterile pustules.^1^, ^2^, ^3^ There are no globally accepted treatment guidelines, and, until recently, there were no approved GPP-specific medications.^2^, ^3^, ^4^ Real-world GPP treatment patterns are poorly characterized. Supplementary material, available via Mendeley at https://doi.org/10.17632/t4fmhr5snv.1.

To describe real-world treatment patterns, this retrospective cohort study (Supplementary Fig 1, available via Mendeley at https://doi.org/10.17632/t4fmhr5snv.1) used claims data from the IBM MarketScan Commercial (MarketScan) and the Optum Clinformatics Data Mart (Optum) databases between October 1, 2015, and March 31, 2020. Patients were aged ≥18 years, newly diagnosed with GPP (International Classification of Diseases 10th revision code L40.1) with ≥1 inpatient or ≥2 outpatient claims 30 to 180 days apart. All analyses were descriptive (Supplementary Appendix I, available via Mendeley at https://doi.org/10.17632/t4fmhr5snv.1).

GPP was rare in both databases (MarketScan, *N* = 502; Optum, *N* = 528 [Supplementary Fig 2 and Table I, available via Mendeley at https://doi.org/10.17632/t4fmhr5snv.1]), with diabetes, anxiety, and plaque psoriasis the most frequent comorbidities (Supplementary Table II, available via Mendeley at https://doi.org/10.17632/t4fmhr5snv.1). Following GPP diagnosis, biologics use was low; topical steroids were the most common first-line treatments (Supplementary Table III, available via Mendeley at https://doi.org/10.17632/t4fmhr5snv.1). There were 151 (MarketScan) and 157 (Optum) patients with 24 months of follow-up with ≥1 dispensing of treatments of interest (ie, typical GPP treatments). At month 1, most patients (MarketScan, 53.6%; Optum, 68.8% [Supplementary Fig 3, *A* and *B*, available via Mendeley at https://doi.org/10.17632/t4fmhr5snv.1]) received none of the treatment classes of interest (Supplementary Table IV, available via Mendeley at https://doi.org/10.17632/t4fmhr5snv.1). Use of treatments of interest increased over time and coincided with increased biologic use (alone and with oral medications) from month 1 to 24 [Supplementary Fig 3, *A* and *B*, available via Mendeley at https://doi.org/10.17632/t4fmhr5snv.1]). Although switching was common in every treatment class, there was no discernible sequence (Fig 1, *A* and *B*). However, patients often transitioned from monotherapy to combination therapy, and the use of biologic monotherapy and biologic-based combination therapies increased over time (Supplementary Appendix I, available via Mendeley at https://doi.org/10.17632/t4fmhr5snv.1).

**Fig 1:**
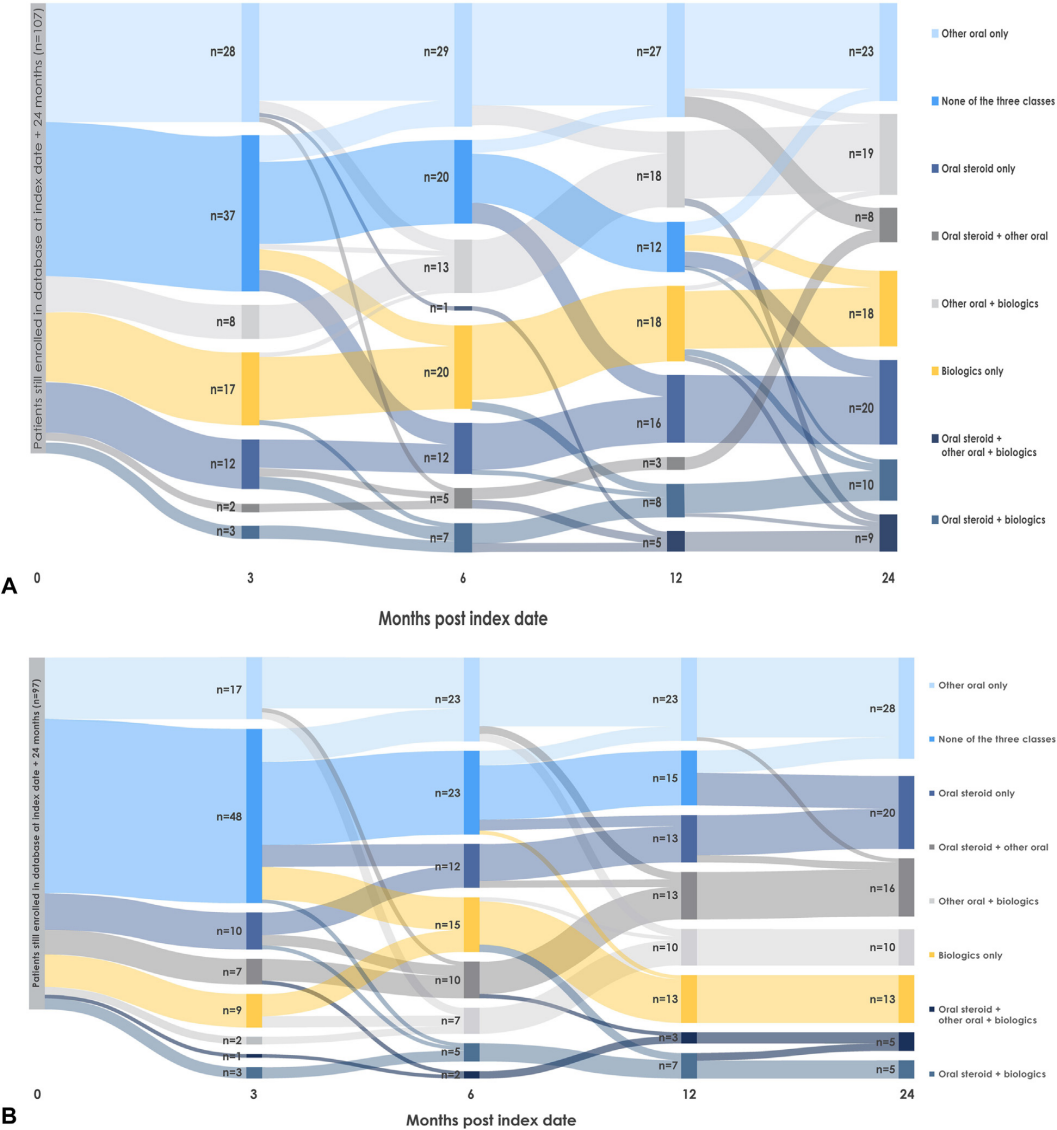
Sankey plots of generalized pustular psoriasis treatment patterns over 24 months in (**A**) the IBM MarketScan Commercial database and (**B**) the Optum Clinformatics Data Mart database cohorts.

The lack of treatment patterns is consistent with the scarcity of approved, GPP-specific treatments and standardized guidelines. Additionally, switching from monotherapy to combination therapy was common, which may reflect a lack of effective treatments. As GPP-specific medications are approved, treatment journeys may become standardized.

The limitations of this study are inherent to the use of claims-based data and should be considered. Utilizing the GPP-specific International Classification of Diseases 10th revision code (L40.1) for analyses relies on it being used correctly and consistently by clinicians; however, the lack of GPP-approved treatments at the time of study may have resulted in miscoding of patients under the general psoriasis code (L40) for treatment purposes. This may explain the lower-than-expected biologic use observed in our results, since miscoded patients would have been excluded from our analysis. However, although the validity of the International Classification of Diseases 10th revision codes for claims-based studies has not been evaluated, previous research has explored GPP disease characteristics in the Optum and MarketScan databases using a similar methodology.^5^

In summary, these findings highlight the challenges of describing real-world treatment patterns in rare, under-recognized diseases, such as GPP, for which there are limited approved treatments. Despite the inherent limitations of claims-based data, GPP treatment patterns are highly variable, indicating the need for more effective approved treatments and evidence-based treatment guidelines.

## Conflicts of interest

Dr Feldman declares receiving research, speaking, and/or consulting support from Eli Lilly, GSK/Stiefel, AbbVie, Janssen, Alvotech, vTv Therapeutics, Bristol Myers Squibb, Samsung, Pfizer, Boehringer Ingelheim, Amgen, Dermavant, Arcutis, Novartis, Novan, UCB, Helsinn, Sun Pharmaceutical Industries, Almirall, Galderma, LEO Pharma, Mylan, Celgene, Valeant, Menlo, Merck, Qurient, Arena Pharmaceuticals, Biocon, Accordant, Argenx, Sanofi, Regeneron, the National Biological Corporation, Caremark, Advance Medical, Suncare Research Laboratories, Informa, UpToDate, and the National Psoriasis Foundation. He is also the founder and majority owner of www.DrScore.com and has stock in Sensal. Author Gao is a former employee of Boehringer Ingelheim. Author Bohn is the founder of Bohn Epidemiology, LLC, and has served as a consultant to Boehringer Ingelheim. Author Gray declares being a consultant to Boehringer Ingelheim and Bohn Epidemiology, LLC. Author Walton and Dr Déruaz-Luyet are employees of Boehringer Ingelheim. Dr Wu declares being an investigator, consultant, or speaker for AbbVie, Almirall, Amgen, Arcutis, Aristea Therapeutics, Bausch Health, Boehringer Ingelheim, Bristol Myers Squibb, Dermavant, DermTech, Dr Reddy's Laboratories, Eli Lilly, EPI Health, Galderma, Janssen, LEO Pharma, Mindera, Novartis, Pfizer, Regeneron, Samsung Bioepis, Sanofi Genzyme, Solius, Sun Pharmaceutical Industries, UCB, and Zerigo Health.
